# Screening and brief interventions for hazardous and harmful alcohol use in primary care: a cluster randomised controlled trial protocol

**DOI:** 10.1186/1471-2458-9-287

**Published:** 2009-08-10

**Authors:** Eileen Kaner, Martin Bland, Paul Cassidy, Simon Coulton, Paolo Deluca, Colin Drummond, Eilish Gilvarry, Christine Godfrey, Nick Heather, Judy Myles, Dorothy Newbury-Birch, Adenekan Oyefeso, Steve Parrott, Katherine Perryman, Tom Phillips, Don Shenker, Jonathan Shepherd

**Affiliations:** 1 Institute of Health and Society, Newcastle University, Newcastle, UK; 2 Department of Health Sciences, University of York, York, UK; 3 Teams Family Practice, Gateshead, UK; 4 Centre for Health Service Studies, University of Kent, Canterbury, UK; 5 Section of alcohol research, Institute of Psychiatry, King's College, London, UK; 6 Northern Regional Drug and Alcohol Services, Newcastle, UK; 7 School of Psychology and Sports Science, Northumbria University, UK; 8 Division of Mental Health, St George's University of London, UK; 9 Humber Mental Health and Teaching NHS Trust, Willerby, UK; 10 Alcohol Concern, London, UK; 11 Violence Research Group, Cardiff University, Cardiff, UK

## Abstract

**Background:**

There have been many randomized controlled trials of screening and brief alcohol intervention in primary care. Most trials have reported positive effects of brief intervention, in terms of reduced alcohol consumption in excessive drinkers. Despite this considerable evidence-base, key questions remain unanswered including: the applicability of the evidence to routine practice; the most efficient strategy for screening patients; and the required intensity of brief intervention in primary care. This pragmatic factorial trial, with cluster randomization of practices, will evaluate the effectiveness and cost-effectiveness of different models of screening to identify hazardous and harmful drinkers in primary care and different intensities of brief intervention to reduce excessive drinking in primary care patients.

**Methods and design:**

GPs and nurses from 24 practices across the North East (n = 12), London and South East (n = 12) of England will be recruited. Practices will be randomly allocated to one of three intervention conditions: a leaflet-only control group (n = 8); brief structured advice (n = 8); and brief lifestyle counselling (n = 8). To test the relative effectiveness of different screening methods all practices will also be randomised to either a universal or targeted screening approach and to use either a modified single item (M-SASQ) or FAST screening tool. Screening randomisation will incorporate stratification by geographical area and intervention condition. During the intervention stage of the trial, practices in each of the three arms will recruit at least 31 hazardous or harmful drinkers who will receive a short baseline assessment followed by brief intervention. Thus there will be a minimum of 744 patients recruited into the trial.

**Discussion:**

The trial will evaluate the impact of screening and brief alcohol intervention in routine practice; thus its findings will be highly relevant to clinicians working in primary care in the UK. There will be an intention to treat analysis of study outcomes at 6 and 12 months after intervention. Analyses will include patient measures (screening result, weekly alcohol consumption, alcohol-related problems, public service use and quality of life) and implementation measures from practice staff (the acceptability and feasibility of different models of brief intervention.) We will also examine organisational factors associated with successful implementation.

**Trial registration:**

Current Controlled Trials ISRCTN06145674.

## Background

Epidemiological data have shown that the majority of alcohol-related problems in a population are not due to individuals with significant alcohol dependence, but to a much larger group of hazardous and harmful drinkers [1]. Hazardous drinking increases the risk of physical or psychological problems whilst harmful drinking is defined by the presence of these symptoms. In the UK, hazardous and harmful drinkers outnumber dependent drinkers by a ratio of 7:1 [1]. Thus it clear that the greatest impact in reducing alcohol-related problems at a population level can be made by reducing alcohol consumption in hazardous and harmful drinkers, rather than by focusing on the most extreme cases of alcohol dependence; this is known as the preventive paradox [2].

Screening and brief alcohol intervention is an example of secondary preventive care [3]. It aims to identify hazardous or harmful drinking at an early stage, before people are consciously aware of (or seeking help for) problems, and then provide advice or counselling to help reduce consumption levels. Primary care is an ideal setting for such activity as this is most people's first point of contact with health services. In addition, primary care deals with a wide range of conditions and aims to prevent as well as treat disease [4]. In the UK, two-thirds of the population visit their GP one or more times each year and 90% attend at least once every five years [5]. In primary care, alcohol screening in routinely presenting cases has shown that one in five patients were drinking at hazardous or harmful levels [6,7]. These patients were consulting GPs and nurses about a wide range of health problems and not about alcohol per se. Thus the large proportion of patients with alcohol-related risk or harm, and the general lack of awareness about the impact of heavy drinking on health, means there is a great potential for screening and brief intervention to help reduce alcohol-related problems in primary care.

### Current evidence on screening and brief intervention

There is a strong evidence-base supporting the effectiveness of brief intervention at reducing alcohol consumption in adults who are not seeking treatment for alcohol-related problems. Numerous systematic reviews and meta-analyses have reported beneficial outcomes of brief intervention, compared to control conditions, in terms of reductions in hazardous and harmful drinking [8-17]. The most recent review of brief interventions in primary care was a Cochrane Collaboration review which included 29 randomized controlled trials of brief alcohol intervention in primary care. This work reported a significant reduction in weekly drinking at one-year follow-up compared to a range of control conditions (such as assessment only, treatment as usual and written information) [17]. The magnitude of this effect was an average reduction of 4–5 standard drinks per week.

Despite the large number of trials in this area, there have been key challenges to the evidence on brief interventions concerning its relevance to routine practice in primary care and its relevance to different types of drinkers. It has been reported that much of the published evidence on brief alcohol intervention has consisted of efficacy trials [18], conducted in tightly controlled research conditions designed to optimize internal validity [19]. Efficacy studies are important in 'proof of concept' contexts where new or early stage treatments are considered. However, if clinicians are to deliver interventions in routine practice, it is necessary to establish that they are effective in clinically relevant contexts. Secondly, its has been observed that participants in brief intervention trials may not be representative of the whole population; the research to date has focused heavily on Caucasian males who are middle-aged and has under-represented minority groups, women and young people [17,20].

In addition, there are key gaps in the evidence-base regarding the cost-effectiveness of brief interventions, the efficiency of screening activity and the ideal format of brief intervention. Regarding the cost-effectiveness of screening and brief intervention in primary care, one US study has reported cost-savings from brief alcohol intervention four years after the intervention was delivered [21]. However, it is necessary to establish if similar cost-savings can be found in different research trials and different country contexts. Also, despite the fact that the vast majority of trials in this field have involved screening to identify patients who require brief intervention, the efficiency and acceptability of screening has been questioned [22,23]. In particular, doubts have been expressed about the strategy of screening all patients (universal screening) and it has been suggested that clinicians should only screen patients with a high likelihood of alcohol-related problems [24]. However, such targeted screening has not been evaluated in terms of either its effectiveness or efficiency in primary care. Lastly, although brief intervention can result in positive changes in excessive drinking, it is not clear if simple structured advice is sufficient for all hazardous and harmful drinkers or if motivationally enhanced counselling provides significant additional benefit to some individuals. The most recent review of brief alcohol intervention found no significant additional benefit of longer compared to shorter brief interventions [17]. However, this meta-regression analysis focused on the length of time clinicians spent delivering brief intervention to patients and not the content of the brief intervention sessions. Simple, structured advice may take less time to deliver and involve less preparatory training but it may also have less impact than motivationally-enhanced counselling. Thus there is a clear need for both an impact and cost-effectiveness analysis of differing intensities of brief alcohol intervention.

In summary, there is a need for more pragmatic research in this field to ensure that the work adequately represents the reality of routine primary care. It is also necessary to ensure that the research trial is located in areas with sufficient diversity to adequately reflect the population of a country. There is a need to evaluate, not just the effectiveness of brief intervention but also its cost-effectiveness at reducing alcohol-related risk and harm. Finally, there is a need to establish the most efficient means of screening patients in primary care so as to minimise the workload for busy clinicians whilst also ensuring that appropriate patients receive brief intervention.

## Aims of the study

To identify the most efficient and acceptable screening approach and tool to detect cases of hazardous and harmful drinking in routine primary care and also to evaluate the clinical impact and cost effectiveness of different models of BI aimed at reducing excessive drinking in routine primary health care.

### Objectives

• To conduct a pragmatic multicentre cluster randomised controlled trial of screening and brief intervention for hazardous and harmful drinkers in primary care in three English regions.

• To identify the optimal method and tool for alcohol screening in routine primary care.

• To compare the effectiveness and cost effectiveness of different models of brief intervention with a control condition in patients with hazardous and harmful alcohol consumption.

• To assess the uptake and use (implementation) of different screening and brief intervention approaches by clinicians in routine primary care.

• To identify attitudinal, practical, skill, resource, and reinforcing factors that may predict successful implementation of screening and brief intervention in primary care.

• To assess the relative impact of the different models of screening and brief intervention on uptake of alcohol services, including an alcohol helpline.

## Methods and design

### Setting

GPs and primary care nurses from 24 practices in three English regions (North East: n = 12, London & South East: n = 12) will be recruited to take part in the study. All primary care practices delivering general medical services in the three regions that do not have current routine screening and brief intervention facilities will be eligible to participate. The study catchment area enables broad population coverage and randomization procedures will ensure that practices cover a range of urban and rural areas, socially deprived and affluent communities, traditional communities with relatively stable populations and more urbanized fluid populations, and culturally mixed populations.

### Design

The trial has a 2 × 2 × 3 nested factorial design encompassing screening approach (targeted versus universal screening), screening method (the Fast Alcohol Screening Test (FAST) [25] or a modified Single Alcohol Screening Questionnaire (M-SASQ)) [26]) and brief intervention intensity (Patient information leaflet (PIL); Brief advice (BA); Brief lifestyle counselling (BLC)). The main advantages of utilising a factorial approach are twofold. First each of the three elements (screening approach, screening tool and intervention) can be analysed independently with sufficient power to make meaningful interpretation of relative effectiveness. Second, the method enables meaningful interpretations of the relative effectiveness of any combination of screening approach, screening tool and intervention modality.

We will recruit 24 general practices from the North East, London and the South East of England. Practices will be randomly assigned to 1 of 2 screening approaches (12 practices in each approach), 1 of 2 screening tools (12 practices using each tool) and 1 of 3 intervention conditions (8 practices in each condition) as shown in Table 1. Since we expect that there may be a difference in uptake of screening between intervention conditions, we expect the recruitment to take varying periods of time in the different conditions.

**Table 1:** SIPS Primary Care trial design

**Intervention condition**		North			London & the South East			
**Screening condition**		Leaflet	Brief Advice	Brief Counselling	Leaflet	Brief Advice	Brief Counselling	Totals

Targeted presentation	FAST	31	31	31	31	31	31	186 (6)
	M-SASQ	31	31	31	31	31	31	186 (6)
Universal presentation	FAST	31	31	31	31	31	31	186 (6)
	M-SASQ	31	31	31	31	31	31	186 (6)

Number of patients per intervention		124	124	124	124	124	124	744
Number of practices per intervention		4	4	4	4	4	4	24

The trial will incorporate cluster randomisation of practices to avoid the risk of contamination. Clinicians trained to deliver 'talk-based' advice or counselling become compromised in their ability to deliver alternative versions of such care; thus it is not practical for individual clinicians to deliver both control and intervention conditions in this trial. Therefore clinicians will consistently deliver a particular version of brief intervention. Cluster randomisation also provides an opportunity to pragmatically evaluate brief interventions in primary care in a way that will allow issues of implementation to be addressed from a practice perspective.

### Study Hypotheses

• Targeted screening methods will result in greater implementation of screening activity than longer more broadly focused (universal) approaches.

• Motivationally-enhanced brief intervention for hazardous and harmful drinkers will be more effective than advice or written information, as follows: BLC > BA > PIL.

• Brief intervention (either advice or counselling) will be more cost effective than merely providing a patient information leaflet.

• Attitudinal, practical, skill, resource, and reinforcing factors will predict screening and brief intervention activity.

### Practice recruitment

Contact with practices will initially be via telephone since this was found to be the most cost-effective method of promoting brief intervention activity in primary care [27,28]. Thereafter a practice visit will be arranged to enable research staff to explain the trial protocol, secure clinician consent to participate in the study and to organise the practice-based training.

### Inclusion criteria

#### Practices

All practices delivering general medical services who have not already instigated screening and brief intervention systems.

#### Patients

Any patient with a positive screening result on FAST or M-SASQ, who is alert and orientated, aged 18 or over, resident within 20 miles, and able to speak, read and write English sufficiently well to complete study questionnaires.

### Exclusion criteria

Patients already involved in an alcohol research study and those seeking help for alcohol problems will be excluded. Any patients who are severely injured, suffering from serious mental health problem and/or are grossly intoxicated will also be excluded from the study. Finally patients with no fixed abode will be excluded from the study.

### Randomisation

Randomisation will be conducted using a secure remote randomisation service. Twenty four allocations will be generated for each of the possible factorial combinations of screening approach (Targeted versus Universal), screening tool (FAST vs M-SASQ) and intervention (PIL vs BA vs BLC). Practices and allocations will be randomly sampled without replacement and paired to generate allocation groups. The randomisation will be stratified by geographical area (north versus south).

### Screening

In order to test the relative acceptability and effectiveness of different screening methods in identifying hazardous and harmful drinkers, we intend to conduct a nested comparison, within our trial, of universal versus targeted screening approaches and of two short screening tools, FAST [25] versus M-SASQ. We were aware from our pilot work that shorter screening instruments are more likely to be adopted in the typical primary care setting than the longer AUDIT. However, it is unclear which tool is most effective in identifying cases of hazardous and harmful drinking in routine primary care settings.

FAST has undergone validity testing in primary care and has been found to be of high sensitivity and specificity [25] and performs well in comparison to the currently recognized 'gold standard', the Alcohol Use Disorders Identification Test or AUDIT [29].

The original SASQ was validated in the USA and asked 'When was the last time you had more than X drinks in one day?' where X = 4 for women and 5 for men [26]. A response of 'monthly', 'weekly' or 'daily or almost daily' is considered a positive screen (1 drink = unit of alcohol). The original values of X were based on US standard drink containing 12 g of alcohol [30]. Since a UK standard drink contains 8 g of alcohol, the equivalent cut-off points in the UK are X = 6 for women and 8 for men. Because SASQ is derived from the third question of AUDIT, we decided to modify the wording so that it accorded with AUDIT since the latter is the key outcome measure in the trial. Hence, the modified M-SASQ asked "How often do you have X or more standard drinks on one occasion?" where X = 6 for women and 8 for men. We validated M-SASQ in pilot work and found that it had a higher sensitivity and specificity than the original SASQ when compared to the AUDIT [29].

Practices will be randomly assigned to either screen all presenting patients (universal screening) or to screen patients presenting with specific linked conditions (targeted screening): hypertension, mental health problems, gastrointestinal problems, injuries and new patient registrations. Within each screening approach, practices will be randomly assigned to use either FAST or M-SASQ. Thus we will have 12 practices in each of the two screening approaches group and 12 practices in each of the two screening tool groups.

Within the universal screening condition, clinicians will record the reason for the presentation in all the patients they screen. Thus we will be able to empirically identify the most common presenting conditions for patients with alcohol-related problems. We will also compare the efficacy of targeted versus universal screening in successfully identifying and providing interventions for the relevant target population. Lastly we will compare the relative efficacy of the two screening tools, and combinations of screening methods and tools, in identifying and delivering alcohol interventions to the relevant target population by considering recruitment rates between screening arms of the trial.

In all conditions, the research team will support participating practices in implementing screening systems tailored to the needs of the practice.

### Consent

Consent to participate will be obtained in a 2-stage process. Primary care staff will initially establish verbal consent to check eligibility to take part, collect some basic demographic information and to be screened. No identifiable information will be collected at this stage. Patients who then are positive on FAST or M-ASQ, as applicable, will have the study explained to them verbally by primary care staff and in writing (using the patient information sheet). Written informed consent will be obtained at this stage which will include permission to give the patient's data and contact details to the research staff, provide the research team with access to the patients' records, and participate in follow up after 6 and 12 months. The research team will then contact the patient within two weeks to thank him/her to take part in the study.

### Interventions

#### Patient Information Leaflet

In the eight practices randomised to the control condition, participating staff will be trained to screen eligible patients for hazardous or harmful drinking. Patients who screen positive and provide consent to participate in the study will complete the baseline questionnaire and then be provided with a patient information leaflet (PIL) . The PIL to be used in this trial is the Department of Health's 'How much is too much? Drinking and you' which contains details of the Drinkline telephone number where the patient can access further information including treatment options for alcohol problems. A sticker with local alcohol services will also be attached to the back cover.

#### Brief advice condition

In the eight practices randomized to deliver Brief Advice, participating staff will be trained to screen eligible patients for hazardous or harmful drinking. Patients who screen positive and provide consent to participate in the study will complete the baseline questionnaire and will then receive up to five minutes of simple structured brief advice from trained staff, using the SIPS Brief Advice tool "Brief advice about alcohol risk" which has been developed for the SIPS programme. It is based on the "How much is too much? Simple Structured Advice intervention tool, developed as part of the UK version of the Drink-Less BI programme [31] from a prototype used as part of a World Health Organisation collaborative study on alcohol screening and brief. Patients in this condition will also receive a Patient Information Leaflet (PIL) at the end of the brief advice.

#### Brief lifestyle counselling

In the eight practices randomized to deliver Brief Lifestyle Counseling, participating staff will be trained to screen eligible patients for hazardous or harmful drinking. Patients who screen positive and provide consent to participate in the study will complete the baseline questionnaire and will then receive up to five minutes of simple structured brief advice from trained ED staff, using the SIPS Brief Advice tool "Brief advice about alcohol risk". Patients in this condition will also receive a Patient Information Leaflet (PIL) at the end of the brief advice. Practice staff will also be trained to deliver a 20 minute brief lifestyle counseling intervention to patients who attend for a subsequent appointment at the practice. The SIPS Brief Lifestyle Counselling (BLC) Tool is based on the "How much is too much?" extended Brief Intervention tool developed as part of the UK version of the Drink-Less BI programme [31] from a prototype used as part of a World Health Organisation collaborative study on alcohol screening and brief intervention.

All intervention tools and protocols are available from the SIPS study website .

#### Training and Support

All primary care staff participating in the trial will be trained to implement alcohol screening and brief intervention according to the trial protocol. The aim of the training is to provide some background information about alcohol related harm, to give an overview of the study protocol, to familiarise staff with the screening tools, structure and scoring procedures, and to inform staff about the procedure for implementing screening and brief intervention at their site. Given the cluster design of the trial, staff will only be introduced to the screening tool and brief intervention condition they have been randomly allocated to. The training is individualised according to the implementation procedure agreed with the local collaborator and senior colleagues in the practice.

A substantial element of the training will involve the understanding of and familiarisation with alcohol units to ensure that the practitioners are fully aware of the alcohol content of different alcoholic drinks so they are able to complete the screening tools accurately. Moreover, as the screening tools refer to standard drinks rather than units when assessing consumption, the training will ensure that staff are aware that a standard drink is one unit and that they are able to convert different drinks into the number of standard drinks e.g. one pint of premium lager equals three standard drinks (or three units). Visual representations of standard drinks as well as several examples of people's drinking patterns will be used to allow trainees to practice calculating units in each drink and to add up the number of standard drinks/alcohol units consumed in order to identify positive cases.

#### Training of staff to deliver brief advice

Participating primary care staff will receive a one hour training session on how to deliver five minutes of brief advice according to the protocol. The aim of the training will be to provide practitioners with the skills necessary to effectively and empathically deliver brief advice about alcohol risk to patients in their practice. The training was developed by the SIPS team to be delivered by an Alcohol Health Worker (AHW). The AHWs in the SIPS team are experienced practitioners in the field of alcohol treatment. They contributed to the development of the training package and have been fully trained to deliver the training to practitioners. The training package is based on a PowerPoint presentation with scripts to standardise delivery. The training sessions are adapted for use in the different experimental conditions in which BA is being delivered.

The session will be presented to small groups of clinicians who will be encouraged to interact with the trainer, ask questions and comment on the content. This will be followed by interactive role play in which the AHW demonstrates the intervention and each practitioner then has an opportunity to practice with a co-worker, observed by the trainer who provides feedback and encouragement. We envisage that training sessions will be delivered to groups of 1–10 practitioners with 3–4 being the typical group size.

#### Training of staff to deliver brief lifestyle counselling

Primary care staff who are suitable to deliver brief lifestyle counselling will receive formal training and supervision on Brief Lifestyle Counselling, in addition to the Brief Advice training. The training will be based upon the previous work of Rollnick et al. [32] in addition to experiences from an earlier trial of screening and brief interventions [33]. The training will comprise four main elements: orientation to the relevant practice, standardised PowerPoint presentation, tape recorded simulated consultations with trained actors and ongoing clinical supervision provided by experienced AHWs.

The simulated consultations will be recorded and rated by three independent clinical assessors. The practitioner will be assessed for adherence to the BLC protocol in addition to their behaviour and skills using the Behaviour Change Counselling Index (BECCI) [34]. Assessors will submit BECCI ratings, comments and supervision points for each consultation. This information will support clinical supervision and training until the practitioner reaches a required standard of practice agreed by an independent clinical assessor.

Support regarding the paperwork for the research will be provided by a research team member, who will also act as the site study coordinator. Research staff and trainers will maintain regular contact with practices throughout the study period, including site visits and telephone support.

### Outcome Measures

#### Staff attitudinal and organisational measures

Participating staff in practices will be surveyed before and after training in each condition of the study to assess attitudinal factors and factors influencing implementation of screening and brief intervention procedures.

Attitudes will be assessed via the shortened Alcohol and Alcohol Problems Perception Questionnaire (SAAPPQ) [35]. A list of all professional staff that can deliver alcohol screening and brief intervention in each study site will be compiled. A self-administered SAAPPQ will be distributed to participating staff on three occasions: pre-training, post-training and post-study. SAAPPQ has five subscales covering role adequacy, role legitimacy, self-esteem, motivation, and work satisfaction. Role adequacy and role legitimacy are concerned with role security, i.e., how individuals perceive the adequacy of their skills and knowledge in relation to problem drinkers and how appropriate it is for them to work with such clients. The subscales relating to self esteem, motivation and work satisfaction, are concerned with worker's therapeutic commitment, i.e., the extent to which they seek to engage drinkers in treatment and the extent that they find the work rewarding on both a professional or personal level [36].

In addition to the SAAPPQ, the post-training and post-study questionnaires will contain a number of semi-structured and open questions developed to elicit information on staff attitudes towards alcohol screening and brief intervention; previous experience of delivering alcohol screening and brief intervention; readiness to undertake these activities; the training needed to conduct screening and brief intervention; the suitability of each site to provide SBI; and potential barriers to effective implementation.

Factors relevant to implementation of screening and brief intervention have been found to be divided into *predisposing*, *enabling *and *reinforcing *factors [37]. Predisposing factors relate to clinicians' willingness to implement screening and brief intervention. Enabling factors are the skills and resources needed to implement screening and brief intervention. Reinforcing factors are visible results, feedback from peers and patients and other factors that encourage continuation of screening and brief intervention.

In the USA, Babor et al. [37] collected data on these factors in two ways: (1) surveys of providers and specialists completed prior to training, after training and at the end of project operations (five items) and (2) independent ratings of site factors made by two research staff during regular technical assistance contacts and site visits (17 items). Inter-rater reliability in Babor et al. [37] was high for all factors (median *r *= 0.70).

In the USA study, predisposing factors were: (1) peer approval for alcohol screening; (2) organizational approval for alcohol screening; (3) the frequency clinicians asked about alcohol consumption; (4) the frequency clinicians educated patients about health risks; (5) the frequency clinicians advised patients with problem drinking to cut down or stop drinking; (6) stable patient membership was based on researcher ratings of whether patient membership was stable or changing; and (7) organisational instability based on ratings of fiscal and management stability. Enabling factors were derived from ratings conducted by two research staff. These factors were: (8) number of clinicians trained at each clinic; (9) doctors' time; (10) nurses' time, (11) receptionists' time; (12) doctors' turnover; (13) nurses' turnover; (14) receptionists' turnover; (15) competing organizational priorities; (16) having an influential site coordinator; (17) involvement of clinic staff in planning; (18) facilitation by computer technology; (19) amount of technical assistance; and (20) successful procedural changes. Two factors classified as reinforcing implementation were also derived from the research staff ratings: (21) organisational support; and (22) financial incentives. An average organizational score was created based on the sum of the 17 items the two researchers rated. This score indicated the total extent of favourable predisposing, enabling and reinforcing factors observed at a given clinic.

In this study we will survey primary care staff before and after training and compare the above factors between different implementation models.

#### System measures

The research team will identify the total number of patients aged over 18 years who attended the practices during the recruitment period, the total number of patients screened, the number screening positive and the number receiving an alcohol intervention in each of the 3 implementation models. This will allow calculation of the overall screening rate, the screen conversion rate (proportion of positive screens) and the intervention rate in the different settings. We will also compare these measures between practices assigned to universal versus targeted screening and the FAST versus M-SASQ screening tools.

Patient re-attendances at practices over the 6 and 12 month follow-up periods will be assessed using computerised records and compared with attendances by participating patients in the 12 months before entry into the study. The sustainability of the screening and intervention approaches will be assessed by examining the extent to which screening and intervention activity continues after the end of the formal study recruitment period.

#### Patient measures

**Figure 1:**
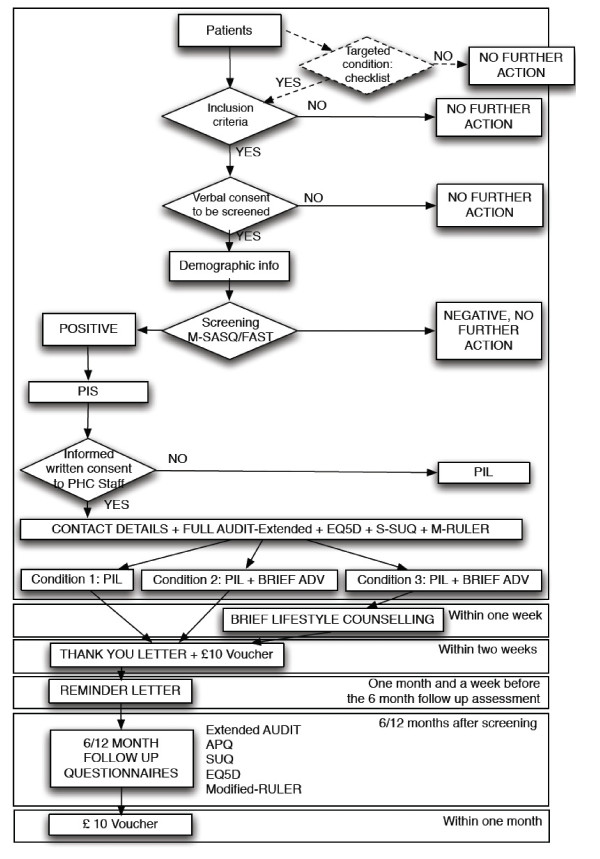
**Measures used with patients and the stage that they are administered**.

A summary of the measures used with patients and the stage that they are administered are summarised in Figure 1.

##### Baseline

Immediately before receiving the initial PIL and/or brief advice intervention, participants will be invited by the primary care staff to provide contact details and complete the Extended AUDIT[29], Euroqol (EQ-5D) [38], a short service use questionnaire (S-SUQ) [39] and modified Readiness Ruler [40]. Participants in the extended intervention will complete the baseline at the same stage as those in other groups.

AUDIT is normally used as a screening test for alcohol use disorders [29]. However in this context the AUDIT will be used as a means of establishing the severity of alcohol use disorders at baseline, in a way that is least intrusive to naturalistic aim of the trial in the primary care setting and as a means of measuring the adequacy of matching between the intervention groups at baseline. The AUDIT contains 10 items to measure alcohol consumption, alcohol problems and dependence over, in this case, the previous 6 months, and the sum of the item scores provides a measure of severity which has been used in several previous studies, allowing comparability with other primary care samples [1]. We felt that the use of more elaborate baseline alcohol consumption measures would interfere with the naturalistic aims of the study and possibly would contribute a form of intervention in itself, so introducing bias into the evaluation of the interventions by reducing the difference between trial interventions. In addition, participants will complete the EQ-5D as a brief 5-item measure of quality of life [38]. Use of health, social criminal justice services and wider societal costs will be measured via a shortened version of Service Use Questionnaire [39] which allows estimation of health care and wider social costs for health economic analysis in the six months prior to intervention. A modified Readiness Ruler [40] containing a zero to ten scale of the extent to which participants think about their drinking as a problem or have addressed this issue will assess participants' motivational state regarding changing their drinking behaviour.

##### Follow-up

At 6 and 12 months after intervention, all patients will be contacted via telephone or post as preferred by research staff who will be blind to their intervention condition. Patients will be offered telephone, postal or face-to-face follow-up as preferred. Researchers will administer the shorter Alcohol Use Disorders Identification Test (Extended-AUDIT) [41]. Alcohol-related problems will be assessed via the brief Alcohol Problems Questionnaire (APQ) [42]. We will also re-administer an extended version of the Service Use Questionnaire SUQ [39], EQ-5D [38]. Patient satisfaction with the advice/help received during the intervention will be assessed using a modified version of the Patient Satisfaction Questionnaire (short form) at 12 months [43].

At follow-up, each patient will also be asked if, and how often, they made use of the Drink-Line telephone number.

All intervention tools and protocols are available from the SIPS study website .

### Financial incentives

Research causes significant disruption to busy practices as well as occupying additional clinical time (consent, assessment, training, data collection procedures etc.). Clinicians are unlikely to participate in a research trial without incentivisation [44] and retention of practices in the trial may also be at risk [45]. However, in this pragmatic trial we were cognisant of the need to evaluate interventions in such a way as to reflect routine primary care. Paying clinicians pro rata for clinical behaviour could undermine our ability to assess implementation outcomes on the basis of acceptability of screening and brief intervention models or how well these have embedded in practice systems. Thus we separated incentives into payment for research participation and for clinical activity.

We will incentivise research participation via the payment of £3,000 to each practice, subject to successful patient recruitment. Payments will be staged, with £1,000 paid at the outset after taking part in the training, £1,000 paid on completion of successful recruitment of participants and £1,000 paid at end of data collection. We hope this schedule of payments will ensure that practices will remain in the trial throughout the study period. A minimum threshold is set of recruiting 31 cases per practice, which would entail screening at least 155 patients per practice since 20% of patients in primary care are likely to be hazardous or harmful drinkers [6]. This will cover the time and disruption caused by participating in the research study. However, practices may stop at this minimum case number.

We will also incentivise screening and brief intervention clinical activity as if this was part of the Quality and Outcomes Framework (QOF) in core general medical services (GMS). We will model the payment on smoking cessation work in the Quality Outcomes Framework (QoF) where points are awarded for building a register of cases and for delivering advice (in 2005, 1 point = £125). At this time, in the coronary heart disease area of QOF, GPs gained 6 points for building a case register (£750) and 10 points for a specific percentage of cases receiving advice (£1,250). We will use practice-level figures (from PCT commissioning colleagues) to scale payments to a per patient fee accordingly (See Table 2).

**Table 2:** Incentives for screening and brief intervention activity

Average practice list size	5,500
Number of eligible adults in the practice	2,250

We assumed 1/3 adults screened	750 cases

Screening fee = 750/750	£1 per patient screened

We assuming 1/5 cases were screen positive	150 cases

Advice fee = 1,250/150	£8 per patient advised*

Counselling is four times longer than advice	31 cases required

Counselling fee	£32 per patient counselled.

* This fee will also be given to control practices delivering the leaflet.

### Participant incentives

Each patient participant will receive a £10 voucher, together with a Thank You letter in the post shortly after completing the baseline research questionnaires and another £10 voucher for completing the 6 and 12 month research follow-up interview.

### Economic evaluation

The economic component of the study will consist of a cost-effectiveness and cost-utility analysis. The study aims to identify, quantify and value resources related to alcohol SBI by clinicians in primary care and the subsequent use of health, social care and criminal justice services by patients following each type of intervention.

Resources utilised in the identification and brief intervention delivery or control condition will be recorded by practice staff involved on an ongoing basis. This will allow the calculation of costs of implementation of different models of screening and brief intervention. Local values will be used to calculate the costs of the interventions, which will include staff costs, premises costs and costs of leaflets and other consumables. In addition, specific training costs for staff will be calculated, in terms of staff time, premises costs and the cost of training materials.

Patients' use of health, social care and criminal justice services will be identified retrospectively using a short SUQ and applying a common set of national unit cost estimates. Patient costs in the 6 month period before SBI can then be compared to cost in the 6 month period after SBI to explore any changes in costs imposed by patients in each group.

The economic analysis will calculate the incremental cost-effectiveness of the control condition with each of the screening and brief intervention conditions under study, using measures of clinical outcome and quality of life responses at 6 and 12 month follow-ups. The use of EQ-5D enables the estimation of Quality Adjusted Life Years (QALYs). Data will be bootstrapped to account for the expected skewness evident in economic cost data [46]. The analysis will include the construction of cost-effectiveness acceptability curves to illustrate the probability that the brief intervention is more cost-effective than usual care, based on different monetary values being attached to QALYs. The use of QALYs follows the recommendations of NICE [47] and enables the value for money afforded by treatment to be compared to a range of other health care interventions. Furthermore, combination of the economic cost data and outcome data with patient data collected in the trial will enable a secondary analysis of various patient characteristics that may influence the cost-effectiveness of the intervention.

### Sample size calculation

The sample size calculation was designed to account primarily for intervention level outcomes. Powering the study in this way will also account statistically for appropriate outcomes for screening approach and screening method. The primary outcome for this study is the proportion of patients who consume alcohol within recommended levels at 6 month follow up. A comprehensive meta-analysis [13] suggested that the difference between brief intervention and control is of the order 13%; that is a 5% reduction in the control group and 18% in the brief intervention group. Detecting a difference of this magnitude at the 5% significance level with 80% power, for a 2-sided test, requires 109 patients in each of the 3 groups and a total of 327. Our experience with other multi-centre randomized controlled trials of interventions for alcohol use disorders suggests that with assiduous follow-up the potential loss to follow-up across groups is of the order 25%. Taking this loss into accounts inflates the sample required to 145 in each group, a total of 435 patients.

The proposed study involves a cluster design and requires a statistical adjustment to account for any potential cluster effect. The literature, and our previous experience of trials in primary care [44], suggest an intra-class correlation coefficient of 0.04 is appropriate. Assuming a cluster size of 31 patients inflated the sample size calculation by a factor of 1.7 requiring 248 patients in each group and a total of 744, with an expectation that at least 558 will be followed up at 12 months.

### Planned analysis

As the study is pragmatic in design, the planned analysis will be by intention to treat. The primary outcome (drinking within or above recommended levels) is dichotomous and so will be analysed with logistic regression adjusting for all known prognostic factors; data will be presented as odds ratios and their corresponding confidence intervals. Secondary analyses will be undertaken using the appropriate method for the outcomes, controlling where appropriate for intake values and other known prognostic variables using analysis of covariance. Due to the nested factorial nature of the study, we will use multi-level modelling to explore potential interactions between each of the three levels nested within the trial (screening approach, screening method and intervention). Practice and patient factors will be utilised as part of regression model to explore possible prognostic factors that impact on outcome. Interaction analysis will explore any possible interactions between practice and patient characteristics and outcome. The efficacy of interventions will be explored with a secondary analysis utilising a per protocol approach; a sub-sample of patients who engaged in their allocated treatment will be utilised in this analysis.

### Ethical and Research Governance Approval

We have obtained Multi-centre ethical approval for the trial (MREC reference number: 06/MRE02/90) plus local agreement from all relevant LRECS. In addition, research governance approval has been granted by all relevant primary care trusts.

### Project Timescales

The trial duration is 30 months and it commenced in April 2008.

## Discussion

Whilst there is a great deal of evidence relating to screening and brief intervention for alcohol problems in primary care, much of this is of questionable relevant to routine clinical practice. The proposed pragmatic trial will provide evidence that will inform primary care practice in both England and outside the UK. In addition to considering the effectiveness and cost-effectiveness of screening and brief intervention at reducing alcohol consumption and its related problems, the trial will also provide important information about implementation issues which are likely to influence future uptake and use of this secondary preventive approach by primary care practitioners.

## Competing interests

The authors declare that they have no competing interests.

## Authors' contributions

This paper is published on behalf of the SIPS programme research group. A full list of the research group members is available at . The study is funded by the Department of Health. The views expressed herein do not necessarily reflect those of the Department of Health or the National Health Service in England and Wales. All of the authors contributed to the design and development of this trial protocol. CD is the Chief Investigator of SIPS and EK is Deputy Chief Investigator and lead for the Primary Care trial. Expertise on clinical aspects of the research was provided for primary care by PC and JM, for nursing practice by TP and for psychiatry CD and EG. Statistical input was provided by SC and MB. Health economics input was provided by CG and SP. Trial conduct and delivery expertise was provided by PD, DNB and KP. Alcohol and policy expertise was provided by AO and DS. Brief intervention expertise was provided by CD, EK, NH and JS. EK wrote the first draft of the paper and all authors contributed to successive drafts. All authors read and approved the final manuscript.

## Pre-publication history

The pre-publication history for this paper can be accessed here:


